# Right Ventricular Outflow Tract Endocarditis: A Very Rare Case and Short View

**DOI:** 10.7759/cureus.89781

**Published:** 2025-08-11

**Authors:** Selman Dumani, Vera Beca, Devis Pellumbi, Stavri Llazo, Edlira Rruci, Daniela Teferici, Altin Veshti

**Affiliations:** 1 Cardiac Surgery, University Hospital Center "Mother Theresa", Tirana, ALB; 2 Nosocomial Infections, University Hospital Obstetrics and Gynecology "Queen Geraldina", Tirana, ALB; 3 Cardiology and Cardiac Surgery, University Hospital Center "Mother Theresa", Tirana, ALB

**Keywords:** infective endocarditis, intravenous drug user endocarditis, mobile vegetation, right ventricular outflow tract endocarditis, tricuspid valve endocarditis

## Abstract

Infective endocarditis involving the right ventricular outflow tract (RVOT) is rare, even among intravenous drug users, and is infrequently encountered by cardiac surgery teams. We report the case of a 30-year-old man with a history of intravenous drug use who presented with a two-month history of fever reaching 39℃. He was initially treated with antibiotics. Transthoracic and transesophageal echocardiography revealed a 2 cm² vegetation on the anterior leaflet of the tricuspid valve, causing severe tricuspid regurgitation, along with a thin, highly mobile, pedunculated mass (1.8-2 cm²) in the RVOT. Surgical intervention was indicated due to the size and mobility of both lesions and the patient's clinical history. The patient underwent tricuspid valve replacement with a No. 33 Epic Supra bioprosthesis and excision of the RVOT mass. Histopathological examination confirmed fibrinous-thrombotic vegetation in both locations. The postoperative course was uneventful. Right-sided infective endocarditis is closely associated with intravenous drug use, with the tricuspid valve being the most commonly affected site. Involvement of the RVOT is rare but should be considered. Surgery remains the gold standard for definitive diagnosis and treatment in such atypical cases and can result in a favorable outcome.

## Introduction

Infective endocarditis involving the right ventricular outflow tract (RVOT) is rare, even among intravenous drug users, and is infrequently encountered by cardiac surgery teams.

Right-sided endocarditis is commonly associated with intravenous drug use. However, it can also occur in other clinical contexts, including right-sided heart catheterization, pacemaker implantation, or congenital heart defects. The incidence of infective endocarditis among intravenous drug users ranges from 1.5 to 20 cases per 1,000 users annually [1]. In the United States, the reported incidence is between 1.5 and 3.3 cases per 1,000 users annually [2]. Acute infection accounts for 60% of all hospital admissions among intravenous drug users, while tricuspid valve endocarditis represents 5-15% of hospitalized cases [2,3]. Additionally, the annual incidence of infective endocarditis among drug users varies between 2% and 5%, contributing to 5-10% of overall mortality in this population [4].

In our case, the patient presented with vegetations on both the RVOT and the tricuspid valve (anterior and septal leaflets). To our knowledge, simultaneous involvement of the tricuspid valve and the RVOT has not been previously reported in the literature.

## Case presentation

A 30-year-old man with a 10-year history of intravenous drug use, under medical treatment supervised by a toxicologist, was admitted to the Infectious Diseases Department where he started intravenous antibiotic therapy. He reported a two-month history of fever reaching 39°C, although he had been afebrile for the past two weeks. On examination, his general condition was stable, with dyspnea on minimal exertion, a blood pressure of 110/70 mmHg, and an oxygen saturation of 97%. Laboratory findings revealed normocytic anemia (hemoglobin 8.6 g/dL; normal reference range 12.1-15.9 g/dL), elevated C-reactive protein (CRP) (6.04 mg/dL; normal reference range <0.5 mg/dL), and a normal white blood cell (WBC) count (5700/μL; normal reference range 4000-10500/μL). Biochemical parameters were within normal limits.

**Figure 1:**
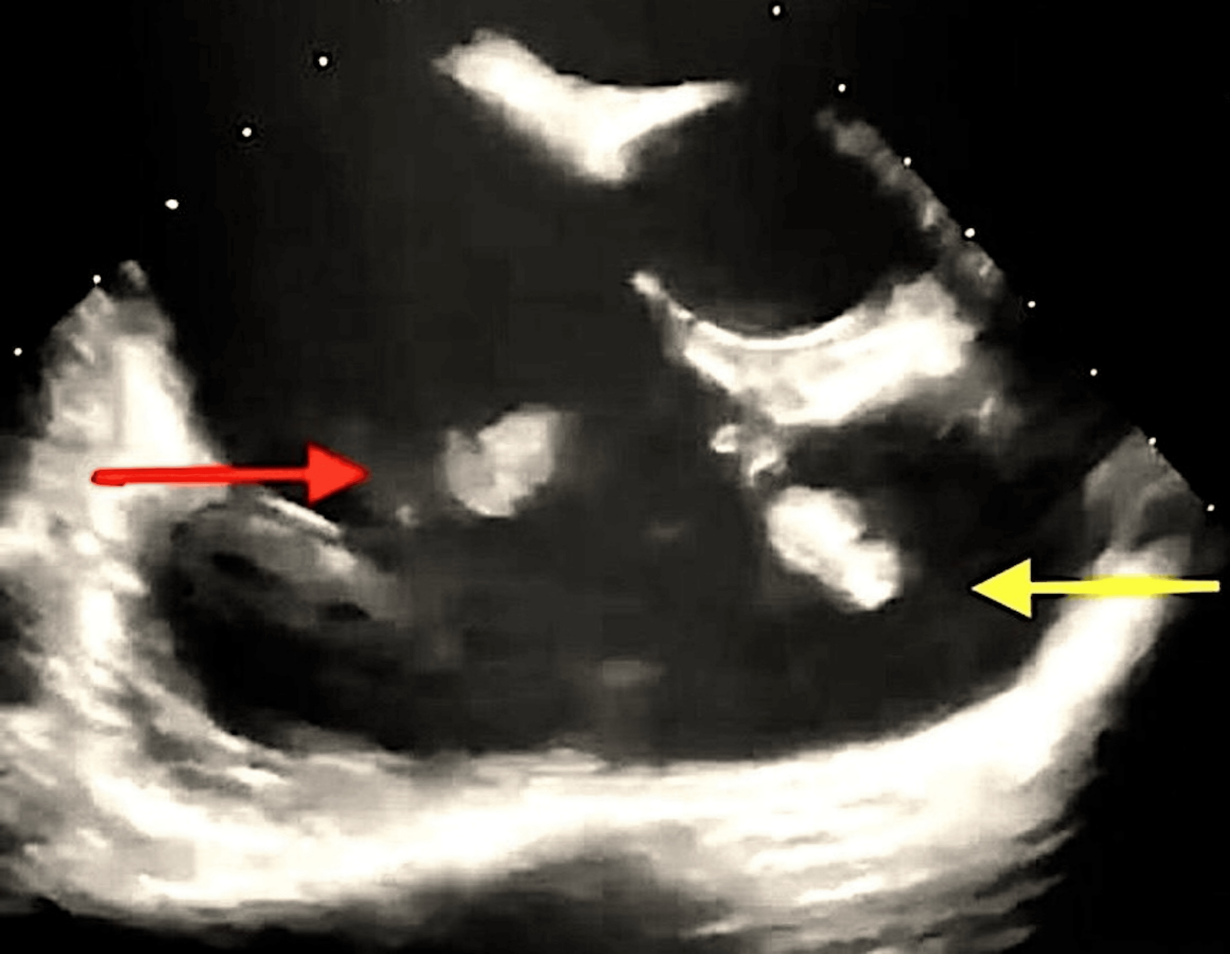
Vegetation on the tricuspid valve (red arrow) and the right ventricular outflow tract (yellow arrow)

Transesophageal echocardiography revealed a 2 cm² vegetation on the anterior leaflet of the tricuspid valve, associated with severe tricuspid regurgitation and a pulmonary artery systolic pressure of 41 mmHg. The right atrium and right ventricle were dilated; however, right ventricular function was preserved. A highly mobile, pedunculated mass measuring approximately 1.8-2 cm^2^ was also observed in the RVOT. The aortic and pulmonary valves were normal, the interatrial septum was intact, the left atrial appendage was free, and the mitral valve was functioning normally (Figure 1).

Under these conditions, surgical intervention was indicated. The surgery was performed through a median sternotomy with aorto-bicaval cannulation. Following aortic cross-clamping and cardioplegic arrest, the right atrium was incised. Intraoperative inspection revealed the complete destruction of the tricuspid valve, with vegetations extending from the anterior to the septal leaflet, making repair unfeasible.

Additionally, a fragile mass with a very small, thin peduncle was visualized in the RVOT. The tricuspid valve, along with the vegetations, was excised and replaced with a No. 33 Epic Supra bioprosthesis. The excised formations were sent for histopathological and microbiological examination. The right atrium was closed, the aorta was subsequently declamped, and the remainder of the operation proceeded without complication.

**Figure 2:**
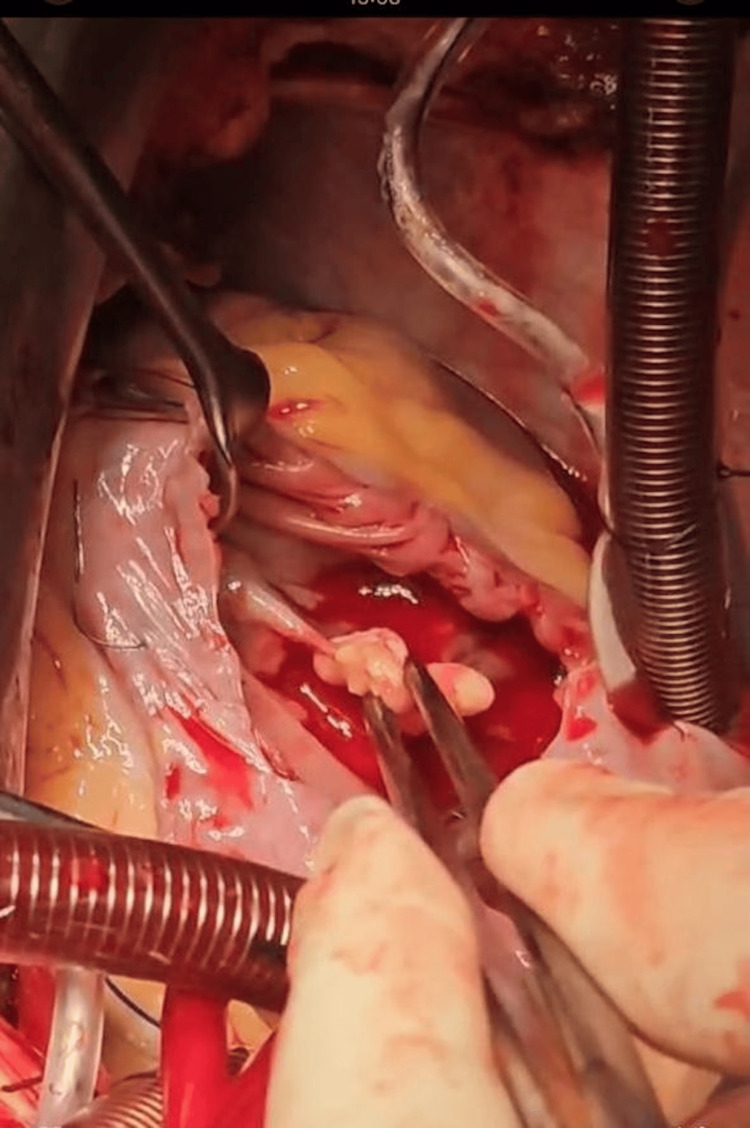
Vegetation attached to the anterior leaflet (between forceps)

**Figure 3:**
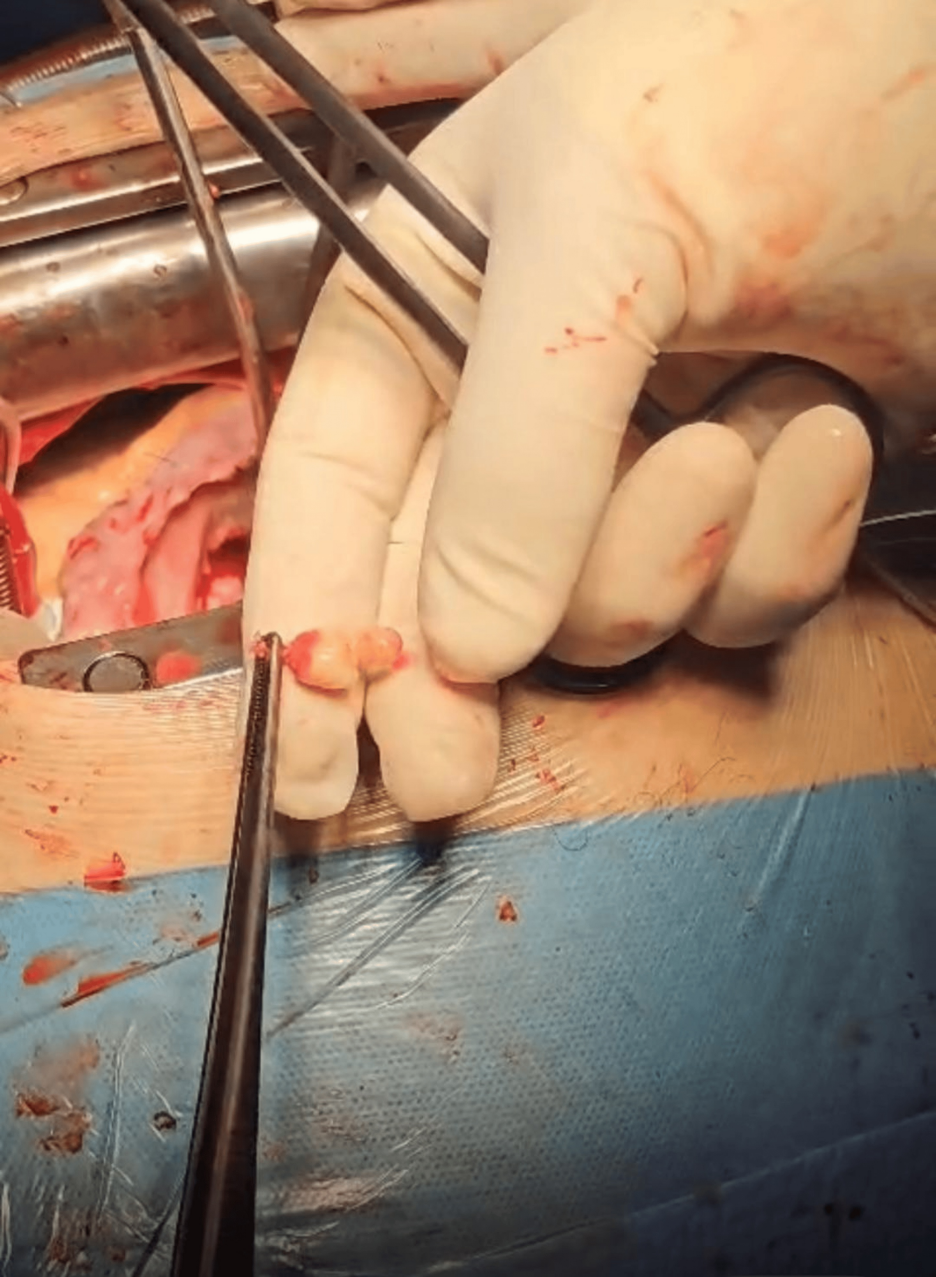
The excised vegetation of the right ventricular outflow tract with a small and very thin peduncle

The photos below were taken in the operating room during intervention (Figures 2-3).

Postoperatively, the patient had an uneventful recovery. At discharge, transthoracic echocardiography demonstrated a normally functioning tricuspid bioprosthesis with a mean gradient of 4 mmHg and no paravalvular leak. Left ventricular function was normal, with no pericardial or pleural effusion.

**Figure 4:**
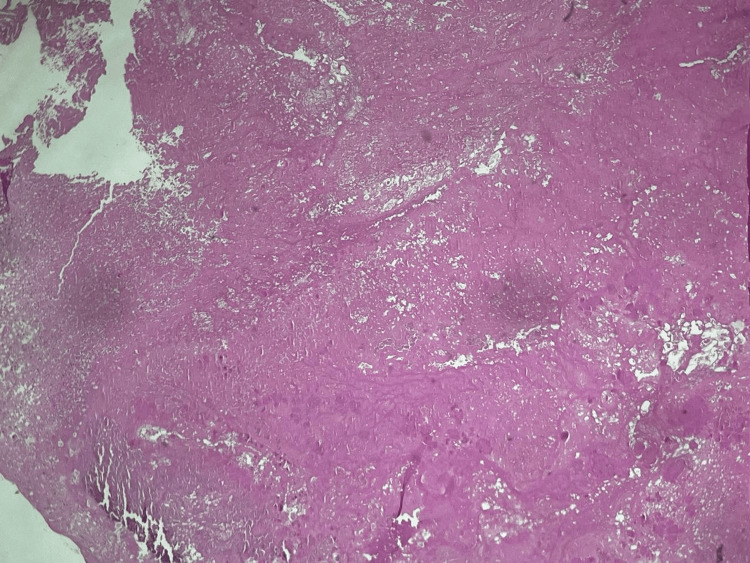
Fibrinous-thrombotic vegetation

Histopathological examination revealed fibrinous-thrombotic vegetations on the tricuspid valve and the RVOT mass (Figure 4). The microbiological culture was sterile. The patient was transferred to the Infectious Diseases Department to continue intravenous antibiotic therapy.

## Discussion

Right-sided endocarditis is commonly associated with intravenous drug use. However, it can also occur in other clinical contexts, including right-sided heart catheterization, pacemaker implantation, or congenital heart defects. The incidence of infective endocarditis among intravenous drug users ranges from 1.5 to 20 cases per 1,000 users annually [1]. In the United States, the reported incidence is between 1.5 and 3.3 cases per 1,000 users annually [2]. Acute infection accounts for 60% of all hospital admissions among intravenous drug users, while tricuspid valve endocarditis represents 5-15% of hospitalized cases [2,3]. Additionally, the annual incidence of infective endocarditis among drug users varies between 2% and 5%, contributing to 5-10% of overall mortality in this population [4]. In the "International Collaboration on Endocarditis-Prospective Cohort Study", which included 2,781 patients with infective endocarditis, 10% had a history of intravenous drug use [5].

In our case, the patient presented with vegetations on both the RVOT and the tricuspid valve (anterior and septal leaflets). To our knowledge, simultaneous involvement of the tricuspid valve and the RVOT has not been previously reported in the literature.

Vegetations are most commonly found on the tricuspid valve but are rarely observed in the RVOT or on the ventricular side of the pulmonary valve [6]. Tricuspid valve involvement has been reported in 90% of right-sided infective endocarditis cases associated with intravenous drug use [7]. However, other right heart structures, including the pulmonary valve, may also be affected. Simultaneous tricuspid and pulmonary valve endocarditis is possible [8]. Isolated pulmonary valve endocarditis is rare, accounting for <2% of all cases, with approximately 70 cases reported between 1979 and 2013 [9].

Sabzi et al. reported a case of isolated RVOT endocarditis due to brucellosis in which the vegetation was attached to the muscular bundle in the right ventricle, with no tricuspid valve involvement. In that case, the vegetation was surgically excised [10].

The most common complications of tricuspid valve endocarditis include valvular regurgitation, abscess formation, and septic pulmonary embolism [11]. In our case, the patient presented with severe valvular regurgitation caused by endocarditis, without evidence of abscess formation or pulmonary complications.

Surgical indications for right-sided infective endocarditis include vegetations >20 mm, persistent bacteremia lasting more than seven days despite antibiotic treatment, right-sided heart failure due to tricuspid valve insufficiency, recurrent pulmonary emboli, and abscess formation, particularly in prosthetic valves [12]. In this patient, the indication for urgent surgery was based on the presence of a relatively large (1.8-2 cm²) pedunculated RVOT mass, severe tricuspid valve destruction, and the patient's age and clinical status. 

Specifically, for tricuspid valve infective endocarditis, 5-40% of patients require surgical intervention [13]. However, surgical management of tricuspid valve endocarditis remains a subject of debate. Valve repair is generally preferred over replacement due to the lower risk of reinfection and reintervention with prosthetic valves [14]. Surgical techniques are classified as either prosthetic (valve replacement or ring annuloplasty) or non-prosthetic (annuloplasty, isolated vegetation excision, or total valve excision) [6]. In patients with tricuspid valve endocarditis related to intravenous drug use, the surgical approach should aim to avoid the use of prosthetic materials and instead focus on vegetation removal and valve repair, as this is associated with better long-term survival [7]. The principle of complete removal of infected tissue and restoration of valve function should be balanced with efforts to minimize the implantation of foreign material. In our case, the anterior leaflet was completely destroyed, and the septal leaflet was partially involved, rendering repair infeasible. Therefore, we performed tricuspid valve replacement using a No. 33 Epic Supra bioprosthesis.

In a study by Yanagawa et al. involving 1,165 patients with tricuspid valve endocarditis, 60% underwent tricuspid valve repair and 40% underwent valve replacement, primarily with bioprosthetic valves. Both approaches showed similar long-term survival; however, valve repair was associated with a longer freedom from recurrent endocarditis, lower reintervention rates, and fewer pacemaker implantations [15]. Similarly, Di Mauro et al. reported no significant difference in short- or long-term survival between tricuspid valve repair and replacement over 25 years among 157 patients with right-sided endocarditis [16]. 

Histopathological examination of the formation is essential to differentiate between thrombus, Löffler endocarditis, and cardiac tumors. Right-sided cardiac tumors are rare and are most commonly located in the right atrium [17]. Myxomas are the most common type of primary cardiac tumor, originating in the right atrium in approximately 3% of cases, and in about 10% of cases, they originate from the tricuspid valvular apparatus, specifically from the chordae tendineae or papillary muscles [18]. Operative mortality for first-time isolated tricuspid valve surgery ranges from 0% to 15% [16]. In our case, the postoperative course was uneventful. 

## Conclusions

Right-sided endocarditis is closely associated with intravenous drug use, with the tricuspid valve being the most commonly affected site. Involvement of the RVOT is exceedingly rare. Early diagnosis and appropriately timed intervention, whether medical or surgical, are critical to achieving optimal outcomes. This case report highlights the need for individualized management strategies to improve patient prognosis. A multidisciplinary team is very important to achieve the main the best results. Surgery remains the gold standard for definitive diagnosis and treatment, even in uncommon locations. It can be performed with very good results.
